# Estimating highest capacity propulsion performance using backward-directed force during walking evaluation for individuals with acquired brain injury

**DOI:** 10.1186/s12984-024-01428-4

**Published:** 2024-08-05

**Authors:** Kelli LaCroix, Lauren Horelka, Clif Hung, David A. Brown

**Affiliations:** 1 MD-PhD Combined Degree Program, Department of Rehabilitation Sciences, University of Texas Medical Branch, 301 University Blvd, Galveston, TX USA; 2 Moody Neurorehabilitation Institute at Galveston, 1528 Post Office St, Galveston, TX USA; 3 School of Health Professions, University of Texas Medical Branch, 301 University Blvd, Galveston, TX USA; 4 Galveston, USA

**Keywords:** English

## Abstract

There are over 5.3 million Americans who face acquired brain injury (ABI)-related disability as well as almost 800,000 who suffer from stroke each year. To improve mobility and quality of life, rehabilitation professionals often focus on walking recovery soon after hospital discharge for ABI. Reduced propulsion capacity (force output of the lower limbs to counteract ground reaction forces) negatively impacts walking ability and complicates recovery during rehabilitation for brain injured people. We describe a method, using backward-directed resistance (BDR) in a robotic-based treadmill device, to allow measurement of maximum walking propulsion force (MWPF) that is not otherwise possible during overground walking assessment. Our objective was to test the construct validity of a maximum walking propulsion force (MWPF) measure that reflects a person’s propulsive strength against applied BDR, while walking on a robotic treadmill-based device for participants with acquired brain injury (ABI). Our study enrolled 14 participants with ABI at an in inpatient rehabilitation in Galveston, TX from 8/1/21 − 4/31/22. The range of weight-adjusted MWPF was 2.6–27.1% body weight (%BW), mean 16.5 ± 8.4%BW, reflecting a wide range of propulsive force capability. The strongest correlation with overground tests was between the 6-minute walk test (6-MWT) distance and the MWPF values (*r* = 0.83, *p* < 0.001) with moderate correlations between the 10-meter walk tests at comfortable (CWS) and fast speeds (FWS). The Five Times Sit-to-Stand (used as a standard clinical measure of functional lower extremity strength) and MWPF tests were poorly correlated (*r* = 0.26, *p* = 0.4). Forward model selection included 6-MWT distance, age, and overground CWS as significant partial predictors of MWPF. We conclude that this novel MWPF measure is a valid representation of maximum propulsive force effort during walking for people post-ABI. Additional research could help determine the impact of interventions designed to increase propulsive force generation during rehabilitation training to improve overground walking performance.

## Introduction

There are over 5.3 million Americans who face brain injury-related disability [1] as well as almost 800,000 who suffer from stroke each year [2]. To improve mobility and quality of life, rehabilitation professionals often focus on walking recovery soon after hospital discharge for such acquired brain injuries (ABI). One goal of physical rehabilitation is to attain optimal functional outcomes such as independent community walking, but therapists are tasked with determining how challenging a training environment should be to match therapy goals with the person’s capacity to realistically achieve them, a concept that been explored by the *challenge point framework* in research [3, 4]. Intensive mobility training, specifically in adults who have had a brain injury, can significantly improve gait speed, balance, and mobility [5], but diminished walking strength due to reduced lower limb power generation and/or poor distribution of lower limb power [6] is a barrier to attaining faster, more appropriate walking speeds. ABI is also associated with hemiparesis and abnormal muscle pattern activation [7] muscle weakness due to gross muscle atrophy, particularly of hip extensors and plantar flexors [8], and neural changes of the motor cortex that result in reduced motor neuron recruitment and rate coding [9]. These factors all contribute to propulsive deficits that are essential to address during rehabilitation.

Studies commonly use the FXSTS as a measure of functional lower extremity strength [10, 11], but biomechanical factors are unique in those who have experienced a head injury. Slower FXSTS times are associated with lower peak whole-body center of mass velocity in a vertical direction, which reflects a decreased ability to perform functional transitional movements and activities such as stair ambulation [12]. However, this ability may not reflect the capability to generate horizontal forward propulsive force during walking. In fact, no studies to date have validated the FXSTS test in people with ABI as a measure of forward propulsion force generation. Overground walking tests such as the 10-Meter Walk Test (comfortable and fast 10MWT) [13] and 6-Minute Walk Test (6-MWT) [14] are the gold standard for measuring walking impairments. These tests provide insight into an individual’s capacity for improvement during rehabilitation and help clinicians set realistic patient goals, track progress, and assess outcomes.

Modern robotic-based treadmills allow the clinician to manipulate the walking force requirements of their clients, which provides a way to analyze forward propulsion force generation in a way that is not feasible in the standard overground environment. Previous research demonstrated a walking assessment based on overcoming horizontal force resistance generated by a robotic assistance treadmill belt in the post-stroke population [15]. This test was performed by applying increased magnitudes of backward horizontal resistance to the treadmill belt while instructing individuals to walk *comfortably* until they reached a level of resistance that prevented them from moving the treadmill belt forward [15, 16]. This test could be further applied to examine upper limits of force generation as an estimate of propulsive force. The KineAssist-MX used in this study can apply backward-directed force to create a precise amount of resistance against forward walking while the participant is walking in the device. A precise amount of resistance can be applied in the opposite direction of walking; when incrementally increased, it is possible to determine a propulsion threshold, referred to as the maximum walking propulsion force (MWPF), for each user.

We developed a MWPF test to examine the forward propulsion generation capability of individuals post-ABI. Our approach was to provide a walking environment with progressive levels of backward-directed resistance (BDR) until the person was no longer able to overcome the forces; this threshold measurement can be used to estimate the potential MWPF that a person could theoretically move at if they were to utilize the propulsive forces during normal (unresisted) walking conditions.

We used the robotic treadmill to explore the use of BDR to determine a MWPF value for participants with ABI. To validate the MWPF measure, we assessed the validity between overground walking tests and MWPF values. We hypothesized the MWPF measure would be positively correlated with overground FWS and the 6-MWT distance. We also hypothesized that MWPF could be used to predict CWS, FWS, and 6-MWT distance overground to potentially explain why some people post-ABI have less endurance compared to others post-ABI. Finally, since we propose that MWPF would be more strongly correlated than the FXSTS with walking performance (e.g., endurance, comfortable walking speed, fast walking speed), we hypothesized there would be a poor correlation between the FXSTS and MWPF measure, reflecting divergent construct validity. Support of these hypotheses would demonstrate that measuring propulsive forces capability can provide useful information about the role of propulsive force generation in walking performance.

## Materials and methods

### Ethics

We obtained study approval from the University of Texas Medical Branch Institutional Review Board (#20-0300.005). Study recruitment and enrollment took place from 8/1/21 − 4/31/22. All study participants gave their informed consent prior to assessment.

### Equipment

This study used the KineAssist-MX (Waukesha, WI), which is a robotic treadmill device with a pelvic harness system that senses horizontal hip forces and allows the participant to walk at their self-intended walking speed or at speeds selected by the administrator while pushing at the center of gravity without creating torque. There is also a ‘deadband value’ that is available for use during progressive-resistive strength training that allows the administrator to set the value of the treadmill belt resistance against stepping forward to walk [17]. The device also allows full freedom of motion and ensures safety during walking tests [18].

### Study population

We assessed 14 participants with ABI who were enrolled at the Moody Neurorehabilitation Institute in Galveston, Texas. Our inclusion criteria were: (1) English-speaking adults at least 18 years of age, (2) ambulatory with/without assistive devices, (3) medically stable (stable cardiovascular status with controlled hypertension and no arrhythmia), (4) willing and cognitively able to provide informed consent. Participants were excluded if they had loss of lower limb, a history of serious cardiac disease (e.g., myocardial infarction), systolic pressure > 140mmHg with diastolic blood pressure > 90mmHg (uncontrolled hypertension), subjects with receptive aphasia or expressive aphasia without a caregiver present to assist, the presence of cerebellar and brainstem deficits, severe cognitive disorder, uncontrolled respiratory disorder, uncontrolled metabolic disorder, major or acute musculoskeletal problems, spasticity management including phenol block or botulinum toxin injections within 4 months of the study, and those with a body weight > 250lbs due to weight restrictions of the robotic device. Clinicians at Moody Neurorehabilitation identified appropriate participants and helped with initial screening. The primary investigator took informed consent at the time of participant screening.

### Data collection

This study was part of a two-part experiment. Part one is a manuscript that is currently under review in which we tested top walking speed capacity. This second part is presented here as a test of maximum walking propulsion force (MWPF) capacity. After giving consent for participation on day 1, participants post-ABI were given a brief 5-minute familiarization period on the robotic treadmill during which they were encouraged to walk at different speeds and purposefully activate the safety harness catch mechanism. Participants then completed standard overground assessments currently recommended by The Academy of Physical Therapy as adult core outcome measurements [19]: (1) 10-meter walk test (10-MWT) at self-selected comfortable walking speed (CWS) and self-selected fastest walking speed (FWS), (2) Five Times Sit-to-Stand (FXSTS) test, and (3) 6-minute walk test (6-MWT). The comfortable and fast self-selected walking speeds were calculated as the average of 3 trials and the fastest of 3 trials, respectively. One trial of the FXSTS and 6-MWT were performed overground. Participants who typically used an assistive device to ambulate independently were allowed to use their device during the 10-MWT and 6-MWT, but did not use any devices during the FXSTS, as participants were instructed to “sit with arms folded across your chest” with the test administrator (physical therapist) standing in front of them to ensure safety. Heart rate and blood pressure were taken immediately before and after the day 1 and 2 walking assessment to ensure that participants had a stable cardiovascular status.

**Fig. 1:**
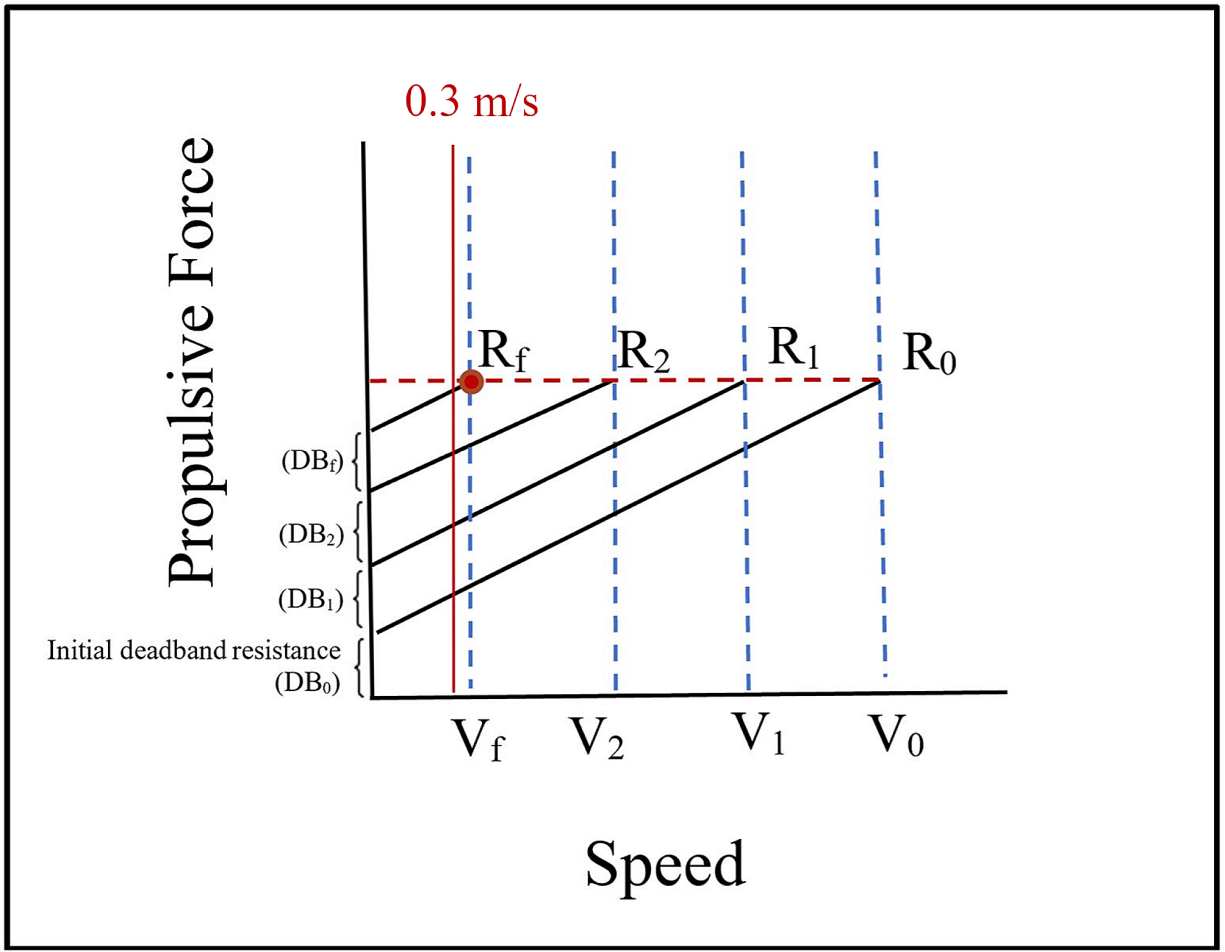
Theoretical relationship between propulsive force and speed occurring with different robotic treadmill resistances used during MWPF test trials. Initial deadband resistance (DB_0_) is programmed automatically into the robotic treadmill as a safety feature. Based on the fastest speed at which the participant travels at each applied deadband resistance (starting at an initial V_0_ and represented by four vertical blue lines), successive deadband levels (DB_1,2_) were calculated until the final, highest deadband resistance applied (DB_f_) at the final speed (V_f_) of ≥ 0.3 m/s. The participant must output a certain amount of propulsive force (horizontal red line along R_0,1,2_) to overcome each level of deadband resistance and maintain a speed of ≥ 0.3 m/s (solid red line). When the participant is no longer to maintain speed ≥ 0.3 m/s, their final tolerated resistance (R_f_) has been determined (red dot)

On day 2, participants were asked to complete assessments in the robotic treadmill-based device: (1) maximum walking propulsion force (MWPF) test (2) a 6-MWT. The 10-MWT at comfortable, self-selected pace was taken from 3 trials using the robotic treadmill and the fast self-selected pace was taken from the fastest speed reached with no treadmill belt resistance added. Per our protocol, 10% body weight support (BWS) was added for participants who were able to perform the 10-MWT overground but were unable to perform the 10-MWT on the robotic treadmill. If the participant was still not able to complete the robotic treadmill 10-MWT with 10% BWS, an additional 10% BWS was added up to the maximum of 40% prior to termination of testing. The MWPF test (see Fig. 1) was performed by incrementally increasing added backward-directed resistance (BDR, measured in lbs) to the treadmill belt, making it harder to overcome and achieve the necessary forward stepping force to move the belt each time. The threshold of maximum walking propulsion force (MWPF) is the highest level of resistance that participants were able to overcome and sustain double stance walking over a brief time, which was at least 5 s per our protocol. At the beginning of the test, participants were instructed to ‘walk as fast as you can’ at a level of no additional resistance (R_0_). The fastest walking speed (V_0_) reached against no resistance was observed on the robot’s digital display and recorded. V_0_ was used to estimate the next, most appropriate, level of added deadband (DB_1_) with the intent of reaching the participant’s maximum resistance threshold as quickly as possible to minimize muscle fatigue. Resistance increased 1lb for every 0.1 m/s walked, based on the fastest speed achieved at the prior level. For example, if an individual walked as fast as they could at 0.6 m/s with 10 lbs treadmill belt resistance, 6 lbs of resistance was added for the next trial performed at 16 lbs of deadband. After that increased resistance was set, we then asked participants to again “walk as fast as you can” at the additional resistance level. Therefore, participants attained lesser and lesser speeds as we added additional resistance. The test continued through subsequent trials until the participant was unable achieve a walking speed ≥ 0.3 m/sec for at least 5 s; each trial lasted no more than approximately 10 s. Between each trial, rest breaks were offered for participant comfort or rest breast were applied to allow participants to return to no more than 10 beats per minute above their baseline heart rate. If a participant was not able to sustain belt speed of at least 0.3 m/sec for 5 s, we divided the previous addition of deadband-lbs in half, to split the difference before attempting the trial again (for the example participant above, we would have reduced the deadband added by half (6 lbs added ÷ 2lb = 3 lbs), so the trial would take place at only 13 lbs). The last successful trial of a speed ≥ 0.3 m/sec (V_f_) was recorded with the corresponding final deadband level (DB_f_) and highest resistance (R_f_) that was overcome by participant-generated propulsive force. The final calculated MWPF used in analysis was adjusted for participant body weight given the strong associations between muscle mass and body size [20]:

(DB_f_ (lbs) / participant body weight (lbs)) * 100 = MWPF (%BW)

One trial of the 6-MWT was performed on the robotic treadmill. Heart rate and blood pressure were taken immediately before and after the day 2 walking tests to ensure that participants had a stable cardiovascular status. See Table 1 for explanation of test variables and walking assessments.

**Table 1:** Walking variables collected

Variable	Abbreviation	Operational Definition	Testing Environment	
			Overground	Robotic Treadmill
**Comfortable Walking Speed**	10-MWT, comfortable	10-Meter Walk Test, comfortable: Overground walking speed when the individual was instructed to “*walk at your normal*,* comfortable speed*”	✓	✓
**Fast Walking Speed**	10-MWT, fast	10-Meter Walk Test, fast: Overground walking speed when the individual was instructed to “*walk as fast as you safely can*”	✓	✓
**Strength**	FXSTS	Five Times Sit-to-Stand: Time (in sec) it takes to stand up and sit down 5 times from a standard chair	✓	N/A
	MWPF	Deadband Resistance/Max Tolerated: Highest resistance (% body weight) reached in a single trial in the robotic device	N/A	✓
**Endurance**	6-MWT	6-Minute Walk Test: Furthest distance walked in 6 min	✓	✓

### Data analysis

This was a repeated measures study design over a short period of 2 days, in first the overground environment and second in the robotic treadmill environment. Each participant was exposed to both testing environments and served as their own control. All variables were summarized using means and standard deviations, or frequencies and percentages, for continuous and discrete variables, respectively.

We examined the association of each overground walking variable (i.e., CWS, FXSTS FWS, and 6-MWT distance) with maximum walking propulsion force (MWPF) attained with the use of BDR, using Pearson’s correlations for these continuous variables. We used the monotrait-heteromethod correlations given the same condition is assessed by different methods (i.e., overground method, robotic treadmill method) [21]. Construct validity is supported when the correlations between the two different methods are high for a single trait to show convergent validity, but correlations between the same methods measuring different traits are low, showing discriminant validity [22]. Significant non-zero slope in linear modeling was used to indicate the presence of an association between two variables, with a perfect association represented by a slope of 1 or -1. Multiple linear regressions were used to examine the relationships between overground walking variables and MWPF determined on the robotic treadmill. Forward model selection was used to identify overground walking variables that significantly contributed to MWPF while controlling for other overground walking variables. All analyses were performed using SAS version 9.4 (SAS Institute, Cary, NC).

## Results

### Participants

The average participant age was 53.1 ± 11.5 years, range 22–62 years. Our sample was 86% male, mean BMI was 26.2 ± 3.3. Walking impairment was mild to severe, as average comfortable walking speed was < 1.0 m/s [23, 24], and mean time since injury was 6.6 ± 3.6 months (range 3-13mo). There were 9 individuals with TBI (2 had hemiplegia, 7 had bilateral deficits) and 5 individuals with CVA (4 had hemiplegia, 1 had bilateral deficits). All participants completed the day 1 and day 2 walking assessments. There were seven participants who used assistive devices (e.g., a variation of a walker, cane) during overground assessments; 2 participants required 10% body weight support and one participant required 30% body weight support (BWS) to complete robotic treadmill walking assessments.

### Resistance threshold with backward-directed resistance (BDR)

**Fig. 2:**
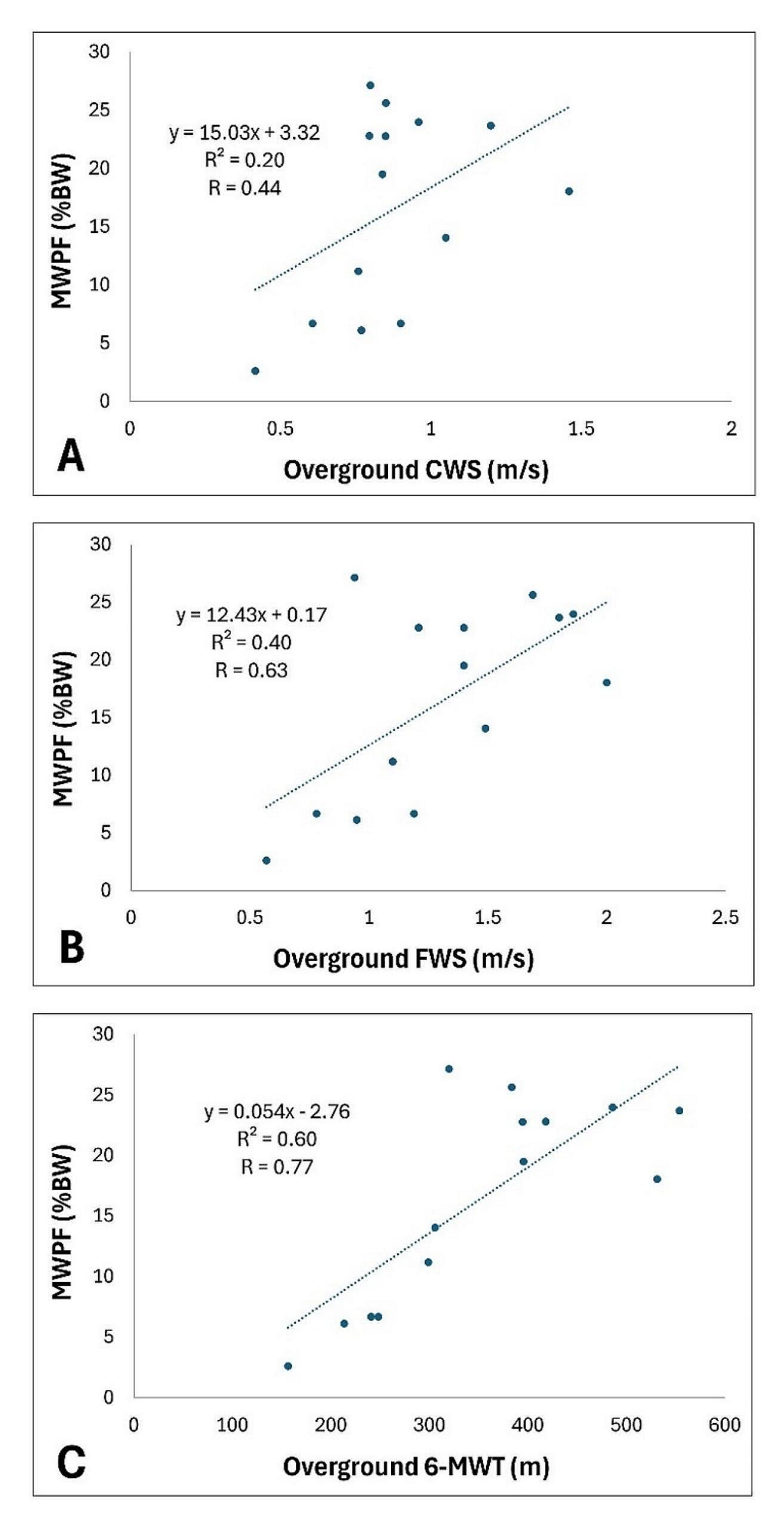
Linear regressions of overground walking tests on maximum walking propulsion force (MWPF) obtained on the robotic treadmill. Linear regression lines are in blue; each blue dot represents a data point from individual subjects. (**A**) Regression of overground comfortable walking speed (CWS) on MWPF. (**B**) Regression of overground fast walking speed (FWS) on MWPF. (**C**) Regression of overground 6-minute walk test (6-MWT) on MWPF

Participants had a mean MWPF of 16.5% total body weight (%BW) (range 2.6–27.1%BW). Figure 2 shows that overground comfortable walking speed, fast speed, and endurance were positively correlated with the MWPF measure in the robotic treadmill-based device. The 6-MWT had a strong correlation with MWPF (*r* = 0.77, *p* = 0.001) and FWS had a moderate correlation with MWPF (*r* = 0.63, *p* = 0.02). There was a poor correlation between MWPF and CWS (*r* = 0.44, *p* = 0.1).

Our overall forward selection model to explain MWPF (R^2^ = 0.76, *p* = 0.002) first included the overground 6-MWT (*b* = 0.07; *r*^*2*^_*partial*_ = 0.60), then age (*b*=-0.23; *r*^*2*^_*partial*_ = 0.09), and finally, overground CWS (*b*=-13.79; *r*^*2*^_*partial*_ = 0.06) as statistically significant predictors of weight-adjusted MWPF. Additional variables of sex, BMI, and time since injury did not significantly contribute to the model and were not included by the forward stepwise selection process.

### The FXSTS correlations with overground walking performance measures and maximum walking propulsion force (MWPF)

The correlations between the FXSTS and overground CWS and FWS were poor (*r* = 0.44, *p* = 0.1 and *r* = 0.32, *p* = 0.3, respectively). There was a poor correlation between 6-MWT distance and FXSTS values (*r* = 0.28, *p* = 0.3). The FXSTS and MWPF values were also not correlated, and the strength of this correlation was poor and statistically insignificant (*r* = 0.26, *p* = 0.4).

## Discussion

The application of backward-directed resistance (BDR) allowed us to assess the measurable limit of forward propulsion force in this study sample. Our study examined maximum walking propulsion force (MWPF) as a measure of walking propulsion strength using BDR applied to the treadmill belt that participants had to use forward walking strength to overcome. We found a strong positive correlation between the between the overground 6-minute walk test (6-MWT) distance and our new MWPF measure as well as a moderate correlation with fast walking speed (FWS), but contrary to our hypothesis, comfortable walking speed (CWS) was poorly correlated.

### 6-minute walk test (6-MWT), age, and comfortable walking speed (CWS) as predictors of maximum walking propulsion force (MWPF)

There is a complex relationship between overground walking predictors and MWPF. The 6-MWT distance explained the highest partial amount of the variability in MWPF (~ 60%) compared to the other overground walking speed measures. Together, the predictors of 6-MWT, age, and CWS may function as a potential surrogate measure of upper walking propulsion limits for those without access to robotic technology to provide safety and accuracy for a MWPF test. An equation that could potentially be used as a surrogate measure to estimate MWPF using the overground measures of the 6-MWT distance (in meters), comfortable walking speed (in meters/second), patient age (in years), and participant weight (in lbs) was:

MWPF (%BW) = [0.11*(6-MWT distance) – 20.11*(CWS) – 0.41*(age) + 29.47]/[(weight)*100]

The equation above explains ~ 76% of the variation in MWPF among our participants. The correlation between actual MWPF scores and predicted scores for each participant based this equation is *r* = 0.87, *p* < 0.001. Overall, this equation indicates an interestingly complex relationship between the 6-MWT distance and CWS. Those who walked farther in 6 min but had a slower comfortable walking speed during the 10-meter walk test were able to generate a higher MWPF. One would expect people with a higher CWS to be able to generate higher MWPF, but it is unclear from this sample’s data why this is not the case. It is possible that individuals who prefer slower walking speeds can reach a higher MWPF due to testing at lower speeds that require less power generation to attain greater force output. Some individuals also choose slower comfortable walking speeds after brain injury compared to non-impaired individuals even though walking at slower speeds is less mechanically efficient, which could be related to alteration of perceived walking speed and individual effort [16]. This could help explain why those who walk slower, potentially at a greater energy expenditure, preserve the ability to generate greater propulsive forces when prompted. The relationship between MWPF and CWS, therefore, warrants further investigation of comfortable walking speed determination in relation to force generation capacity. Our model fit equation should also be further validated in an independent sample to improve generalizability in this patient population. Further studies are also needed to determine whether the relationships among these variables and MWPF are confounded by other factors that could be related to body structure/function, activities, participation, or even personal/environmental factors.

Results from this study show that people who can generate higher MWPF are able to walk farther during a 6-MWT, but the reasoning behind this relationship may not be immediately intuitive. One explanation for increased strength requirements of walking farther distances could be the necessity to maintain speeds over a prolonged time to combat muscle fatigue experienced during tests of endurance. Our study minimized muscle fatigue by limiting the overall number of trials (using participant fastest walking speeds at each resistance level to estimate subsequent resistance levels) as well as limiting each trial to a brief 5–10 s period. As previously mentioned, after ABI, muscle fatigue requires increased motor recruitment and increased neuronal rate coding [9]; those who are weaker and unable to accommodate these increased requirements are expected to slow their walking speed as muscles necessary for walking, primarily the plantar flexors and hip extensors [8], fatigue. After ABI, people may also struggle with appropriate pacing during the 6-MWT due to impaired sensory input/integration or executive functioning that would lead to even greater effects of fatigue compared to people without injury [25]. Evidence of this phenomenon could be determined from a future study in which participants post-ABI as well as non-impaired participants are monitored over the course of a 6-MWT (distance, perceived exertion, and actual metabolic output could be recorded each minute) for comparison of walking habits over an extended period. A future study aimed at examining the effect of an individual’s resistance to muscle fatigue could also better explore the complex relationships between MWPF and overground walking test measures.

Finally, we found that age was selected into our MWPF model. Sarcopenia refers to an age-related loss of muscle mass that results in decreased muscle strength [25]. As people age, particularly after the 5th decade of life, both isometric (muscle tension from contraction, but no movement) and concentric (active muscle contraction leading to movement) muscle strength decline at various rates due to normal aging as well as other age-related factors such as inactivity and multiple comorbidities [26]. Age has also been suggested to play a role in postural stability, particularly in a treadmill environment, due to visual difficulty and decreased ability to learn and adapt to new environments [27]. Our findings were consistent with this association between age and decreased lower extremity strength [28]; those who are older tend to have a decreased ability to generate walking propulsion force. This is largely suspected to be due to decreased muscle mass (i.e., sarcopenia), but could also partially due to difficulty with performing the MWPF test in an unfamiliar environment that requires some learning and adaptation.

### 5 times sit-to-stand (FXSTS) and walking propulsion force

Our hypothesis that the FXSTS measure is a poor measure of MWPF in the robotic treadmill was supported, as the FXSTS and our MWPF measure of propulsion force limits showed a high degree of construct-related divergent validity. Although the FXSTS is a commonly used measure of functional strength for those with disability, this study indicates that it is not a useful measure of walking strength. The FXSTS has been used to measure lower extremity strength and balance [11], but biomechanical factors for those who have experienced a head injury have shown slower FXSTS times are associated with lower peak whole-body center of mass and this test reflects ability to perform a functional transitional movement and activities such as stair ambulation [12]. A study by Zablotny et al. examined failed FXSTS trials for an individual with traumatic brain injury and found that decreased whole-body center of mass vertical velocity due to insufficient knee extension angular velocity explained unsuccessful rising attempts [29], which is not the mechanism of hip extension and plantar flexion thought to contribute to forward walking propulsion [30, 31]. It has also been shown that the FXSTS in older adults not only depends on strength, but other factors such as sensation, balance, and psychological status [11]. There is no standardized clinical measure of forward propulsion force generation, therefore it was of importance to examine the only overground measure currently recommended for clinical practice that attempts to quantify functional lower extremity strength [10]. We wanted to determine if the FXSTS is a valid measure of MWPF in our study population given the test differences in task specificity. The MWPF test presented here also has a considerably higher degree of face validity: the MWPF test is performed during flat surface walking​, the generated horizontal propulsion forces are measured during this test, and the MWPF test, by design, only stops when robotic treadmill deadband forces can no longer be overcome. This finding provides evidence that the FXSTS measure, which has been used in previous research to quantify lower extremity muscle strength [32, 33], is not an appropriate measure to determine someone’s capacity for walking propulsion, particularly after brain injury.

### Study limitations

This current study is not without limitations. Although there was a brief familiarization period with the robotic device, it was still a very new experience for our participants. In addition, visuospatial feedback (e.g., proprioception, visual flow) differs in the robotic treadmill vs. walking overground [34]. Multiple practice trials on a separate day prior to testing could help account for motor learning needs, which may have negatively skewed the MWPF measurement. This would be particularly impactful if participants perform better as they feel more comfortable using the device over a more prolonged period. Also, there were 7 of 14 total participants who used their usual assistive device in the form of a walker or single-point cane during the overground 10-meter walk tests and 6-minute walk test, which was meant to provide increased safety during these measures. Although each participant served as their own control for comparison between overground and robotic treadmill measures, is unclear how the association between overground walking speed/endurance and MWPF measures were affected. Future studies with a larger sample size should be performed to better power an analysis of the effect of using an assistive device during these tests. While this study used core measurements recommended by The Academy of Physical Therapy, another assessment to consider in future studies could be the Timed Up and Go, which in theory may be more closely related to walking propulsion and gait assessment as the test requires both walking a short distance and standing/sitting in a chair [35].

## Conclusion

The application of backward-directed resistance (BDR) allowed for the methodical estimation of maximum propulsive force effort, representing an increased capability within our sample. This maximum walking propulsion force (MWPF) measure reflects a higher degree of convergent and face validity than the Five Times Sit-to-Stand (FXSTS) test; theoretical calculations on top propulsive ability may be greater than overground tests can determine. Additional research could help determine the impact of interventions designed to increase propulsive force generation during rehabilitation training to improve overground walking performance.

## Data Availability

The datasets used and/or analyzed during the current study are available from the corresponding author on reasonable request.
